# Probable fatal drug interaction between intravenous fenretinide, ceftriaxone, and acetaminophen: a case report from a New Approaches to Neuroblastoma (NANT) Phase I study

**DOI:** 10.1186/1756-0500-7-256

**Published:** 2014-04-23

**Authors:** Min H Kang, Judith G Villablanca, Julia L Glade Bender, Katherine K Matthay, Susan Groshen, Richard Sposto, Scarlett Czarnecki, Matthew M Ames, C Patrick Reynolds, Araz Marachelian, Barry J Maurer

**Affiliations:** 1 Cancer Center and Departments of Cell Biology and Biochemistry, Pharmacology and Neuroscience, Pediatrics, and Internal Medicine, School of Medicine, Texas Tech University Health Sciences Center, 3601 4th Street, STOP 9445 79430 Lubbock, TX, USA; 2 Department of Pediatrics, University of Southern California, 4650 Sunset Blvd, 90027 Los Angeles, CA, USA; 3 Department of Pediatric Hematology & Oncology, Columbia University, 161 Fort Washington Ave, 10032 New York, NY, USA; 4 Division of Pediatric Hematology-Oncology, University of California San Francisco, 505 Parnassus Ave M647, 94143 San Francisco, CA, USA; 5 Department of Biostatistics, School of Medicine, University of Southern California, 1441 Eastlake Ave, 90089 Los Angeles, CA, USA; 6 NANT Operations Center, Children’s Hospital Los Angeles, 4650 Sunset Blvd, 90027 Los Angeles, CA, USA; 7 Department of Molecular Pharmacology and Experimental Therapeutics, Mayo Clinic, 17778 East North Shore Lane, Bayview, 83803 Idaho, USA

**Keywords:** Ceftriaxone, Fenretinide, Acetaminophen, Drug interaction, Biliary sludge, Fulminant hepatic failure

## Abstract

**Background:**

Patients with relapsed/refractory stage 4 high-risk neuroblastoma were enrolled on a phase I study (NANT2004-03) of intravenous fenretinide emulsion. Pharmacokinetic samples were collected during and after the infusion, and the levels were measured using an HPLC system. A likely case of a fatal drug interaction between fenretinide, ceftriaxone, and acetaminophen is described, including the pharmacokinetics of fenretinide, laboratory data, and post-mortem autopsy in a pediatric neuroblastoma patient treated on this study.

**Case presentation:**

On Day 4 of a scheduled 5-day-infusion of intravenous fenretinide, the patient developed a fever, acetaminophen was started, ceftriaxone initiated for possible bacteremia, and fenretinide level doubled from 56 to 110 μM. Over the next three days, although blood cultures remained negative, the patient’s condition deteriorated rapidly. Acute liver failure was diagnosed on Day 7, and the patient expired on Day 20 of fulminant hepatic failure with associated renal, cardiac, and hemorrhagic/coagulation toxicities. Autopsy showed extensive hemorrhagic necrosis of the liver, marked bile duct proliferation, and abundant hemosiderin, consistent with cholestasis and drug toxicity.

**Conclusions:**

After extensive review of patient data, the clinical course, and the literature, we conclude that observed hepatic toxicity was likely due to a drug interaction between fenretinide and concomitant ceftriaxone and acetaminophen. None of the other 16 patients treated on this study experienced significant hepatic toxicity. Although the prevalence of cholestasis with ceftriaxone usage is relatively high, the potential drug interaction with these concomitant medications has not been previously reported. Concomitant use of fenretinide, ceftriaxone, and acetaminophen should be avoided.

## Background

Ceftriaxone is a widely-used, semi-synthetic third generation cephalosporin antibiotic noted for its broad activity spectrum, long plasma half-life, and relative paucity of side effects. However, the association of ceftriaxone and biliary ‘sludging’ (pseudolithiasis) is well documented. [1-4]. Ceftriaxone is present in bile at 20- to 150-fold higher concentrations than serum, is minimally metabolized, and is excreted as a divalent anion that is calcium sensitive [4]. It is speculated that the pseudolithiasis results from the combination of a hepatic effect, wherein the liver secretes a biochemically abnormal bile, and a gall-bladder effect, which provides the environment for anion precipitation [4]. With rapid onset and disappearance, ceftriaxone-calcium sludging is generally asymptomatic, but can result in frank cholelithiasis (i.e., gallstones) [4]. The incidence of biliary sludging from ceftriaxone ranges from 25% to 46%, serious complications rarely occur [3].

Liver toxicity from acetaminophen, a common pediatric antipyretic/analgesic is the most common cause of acute liver failure in the USA [5]. Recommended pediatric acetaminophen dosing is 10–15 mg/kg every 4–6 hours with daily maximum dose of 4 grams. Under normal hepatic clearance, acetaminophen is principally conjugated to a nontoxic glucuronide or sulfate. However, under conditions of acetaminophen excess or disturbed metabolism, cytochrome P450 2E1 can generate a reactive acetaminophen intermediate, N-acetyl-p-benzoquinone imine (NAPQI), which interacts with proteins and nucleic acids to damage the liver [6]. As NAPQI is conjugated to glutathione and renally excreted, acetaminophen overdose overwhelms hepatic glutathione capacity leading to accumulation of unconjugated NAPQI and hepatotoxicity [6]. Children appear to be less susceptible to acetaminophen toxicity than adults because they have less capacity for oxidative metabolism [7].

Fenretinide (N-(4-hydroxyphenyl)retinamide; 4-HPR) is a synthetic cytotoxic retinoid with chemopreventative activity in animal models [8], and cytotoxic activity in a variety of human cancer cell lines *in vitro *[9-11]. Oral capsular fenretinide has a plasma half-life of ~15-20 hours [12]. Fenretinide is hepatically cleared, at least in part, by cytochrome CYP3A4-dependent metabolism, and glucuronidation by uridine 5’-diphospho-glucoronylosyl transferases (UGTs). Three pediatric cancer clinical trials of the capsule formulation (largely in neuroblastoma) reported minimal toxicity [12,13]. Grade 3–4 hepatic toxicities included hypoalbuminemia, and elevations of AST, ALT, bilirubin, and alkaline phosphatase, which resolved without sequelae. One patient developed fatal hepatic failure determined on autopsy to be due to massive tumor infiltration of the liver and felt to be unrelated to fenretinide. However, tumor responses in these trials were limited, possibly due to poor bioavailability of the capsular formulation. Novel powder and intravenous formulations have demonstrated improved bioavailability as evidenced by higher plasma levels [14,15]. A phase I study of the LXS powdered formulation achieved plasma levels 2–6 fold higher than the capsular formulation and four complete responses [14]. Hepatic toxicity was limited to grade 3 AST/ALT and Grade 4 elevation of alkaline phosphatase which resolved without sequelae.

The New Approaches to Neuroblastoma Therapy (NANT) consortium conducted a Phase I trial of an intravenous fenretinide emulsion formulation (IND#: 70058). We report a case of fatal hepatic failure during course one of therapy. After a thorough review of the clinical course, concomitant medications, laboratory data, and fenretinide plasma levels, we conclude that the event likely derived from an unexpected multi-drug interaction between fenretinide and ceftriaxone and acetaminophen.

## Case presentation

The NANT 2004–03 Phase I study (ClinicalTrials.gov Identifier NCT00646230) escalated the dose of intravenous (IV) fenretinide (given for five days as a continuous infusion and repeated every 21 days) using a standard 3 + 3 design to determine the maximum tolerated dose (MTD). Toxicities were graded using the Common Terminology Criteria for Adverse Events (CTCAE), version 3.0. (http://ctep.cancer.gov). Eligible patients were ≤ 30 years of age with relapsed/refractory high risk neuroblastoma. Organ function required included bilirubin, AST, ALT, and creatinine ≤ 1.5 × normal. Plasma fenretinide levels were obtained at hours 0, 6, 12, 24, 36, 48, 72, 96, 120 (end of infusion), then +2 hours and +48 hours after the end of infusion, protected from light, immediately frozen, and analyzed using an HPLC method [16]. The study was terminated prior to determination of the MTD due to inadequate drug supply after the enrollment of 17 patients. Table 1 summarizes dose levels tested, dose limiting toxicities, and all hepatic toxicities.

**Table 1:** Dose limiting toxicity and maximum grade of all hepatic toxicities for all 15 patients enrolled on NANT 2004–04

**Total dose of IV fenretinide**	**# patients enrolled (# evaluable for dose escalation)**	**Patient Age at study entry (years)**	**Fenretinide peak level Course 1 (μM)**	**Dose Limiting Toxicity**	**Total number courses/Hepatic toxicities (maximum grade (Gr) across all courses received )**	**Ceftriaxone during or after fenretinide**	**Acetaminophen during or after fenretinide**
Level 1: 640 mg/m^2^/day	3 (3)	7.9	30.5	None	6 courses: Gr 2 AST, Gr 2 hypoalbuminemia, Gr 1 hyperbilirubinemia	3 days during Course 6	
		8.5	17.2	None	1 course: Gr 1 hypoalbuminemia, Gr 1 AST		1 dose 2 days after Course 1
		24	30.1	None	2 courses; none		
Level 2: 770 mg/m^2^/day	3 (3)	4.2	40.7	None	30 courses: Gr. 3 ALT, Gr 3 AST, Gr. 2 hypoalbuminemia, Gr 1 alkaline phosphatase. Got dose level 1 for courses 26–30 due to delayed platelet recovery course 25.		20 doses starting after completion of Course 14
		4.4	70.2	None	4 courses; Grade 1 AST		
		5	39.4	Gr 3 hypoalbuminemia*	1 course: Gr 3 AST, Gr 3 hypoalbuminemia*		
Level 3: 925 mg/m^2^/day	7 ( 6 )	6.7	36.3	None	2 courses: Gr. 1 ALT, Gr 1 AST, Gr. 1 hypoalbuminemia		4 doses during Course 3
		5.2	38.4	None	6 courses; Gr 1 AST, Gr 1 hypoalbuminemia, Gr 2 hyperbilirubinemia	2 days during Course 1	
		9.7	60.7	Gr 4 hypertriglyceridemia due to error in drug infusion	1 course: Not evaluable for dose escalation due to error in drug infusion with 24 hour dose given over 12 hours; Gr 1 hypoalbuminemia, Gr 2 hyperbilirubinemia		
		11.4	33.0	Pseudotumor cerebri	1 course: Gr 3 ALT, Gr 2 AST		
		11	29.1	None	1 course; Gr 1 ALT, Gr 2 AST, Gr 1 hypoalbuminemia		
		5.3	23.7	None	2 courses: Gr 1 AST		
		12.6	45.0	None	2 courses; Gr 1 ALT, Gr 2 AST		
Level 4: 1110 mg/m^2^/day	2 (2)	5.5	83.3	Gr 4 epistaxis related to multi organism non-neutropenic bacteremia during course 5	5 courses: Gr 1 ALT, Gr 2 AST, Gr 2 hypoalbuminemia, Gr 1 alkaline phosphatase	4 days starting 9 days after completion of Course 5	
		7.2	110.9	Fatal hepatic failure, renal failure, and hypotension**	1 course (subject of this case report): Gr 5 liver dysfunction/failure (clinical), Gr 4 ALT, Gr 4 AST, Gr 4 hyperbilirubinemia; Gr 3 hypoalbuminemia; Gr 1 alkaline phosphatase	3 days starting 4^th^ day of the infusion	9 doses starting 4^th^ day of the infusion
Level 3a: 925 mg/m^2^/day	2 (0)	6.7	14.17#	None	2 courses: Gr 1 ALT/Gr 1 AST		
		7.8	21.93#	None	5 courses:Gr 1 AST, Gr 1 hypalbuminemia, Gr 1 alkaline phosphatase		

*Patient enrolled with Grade 1 hypoalbuminemia, resolved from asymptomatic Grade 3 to Grade 2 by day 27 of course; Grade 1 by Day 31 (definition of DLT was resolution to Grade 1 by Day 28). Dose level not expanded since not considered clinically significant & protocol amended to exclude metabolic abnormalities as DLT. ** Subsequent Gr 4 acidosis, colitis, disseminated intravascular coagulation, enteritis, hypoxia, and hemorrhage; Gr 3 encephalitis as described in the text. # Both patients had course 1 infusion interrupted for hypertriglyceridemia and dose reduced by 50% to complete course 1 and for subsequent courses.

The patient was a seven year-old male diagnosed with stage 4 high-risk neuroblastoma in October 2007, who received induction according to the ANBL02P1 regimen (NCT00070200) which included cyclophosphamide, topotecan, cisplatin, etoposide, doxorubicin, and vincristine [17] followed by myeloablative therapy with I^131^-MIBG, carboplatin, etoposide, and melphalan with autologous purged hematopoietic stem cell transplant in May 2008 on NANT Protocol 2001–02 (NCT00253435). He received 2160 cGy local radiation to the right adrenal primary and distal femurs in July 2008, two courses of 3 F8 anti-GD2 antibody, nine courses of isotretinoin, and an additional 1440 cGy radiation to a single persistent skull metastasis in July 2009. He then received five courses of irinotecan and temozolomide for refractory bone metastases September-December, 2009. Bone marrow exam done January 2010 showed recurrent tumor, with no other relapse sites by CT or MIBG scans. No additional anti-cancer therapy was received until the intravenous fenretinide in February 2010 (assigned dose 1110 mg/m^2^/day as a continuous infusion for 120 hours).

The patient tolerated therapy without adverse events until Day 4 when he developed fever (38.2°C) without localizing symptoms. Acetaminophen (10 mg/kg) every 4 hours was initiated, and increased to 15 mg/kg beginning with second dose. After blood cultures, intravenous ceftriaxone was initiated. On the same day, temperature increased to 39.4°C, despite acetaminophen and cooling measures, with Grade 1 abdominal pain (had not stooled in five days) and Grade 2 headache. Abdominal exam was soft with normal bowel sounds. Neurologic exam was normal and fundi without papilledema. The headache was assessed as likely due to fever/dehydration but pseudotumor cerebri from fenretinide could not be ruled out, so infusion was stopped on Day 5 at Hour +119 of the scheduled 120 hour infusion. Intravenous fluids were given for poor oral intake and decreased urine output. Acetaminophen and one dose of ibuprofen were given for fever management. Two doses of ibuprofen were administered Day 5. On the night of Day 5, headache improved to Grade 1, but intermittent fever persisted and abdominal pain increased to Grade 3. Abdominal plain film was consistent with constipation.

Day 4 lab studies included an elevated LDH of 1241 U/L (797 baseline), bilirubin 0.6 mg/dl (0.2 mg/dl baseline), AST 57 U/L (39 U/L baseline), ALT 25 U/L (17 U/L baseline). No labs were performed on the morning of Day 5. On Day 5, the patient became hypotensive despite fluid support and required pressor support (dopamine). Oxygen saturation was 90% on room air but normal on mask oxygen. Blood bacterial cultures from Days 4, 5, 6 and 7 and fungal cultures from Day 6 were negative. On Day 6, AST further elevated to 698 U/L, ALT elevated to 259 U/L, creatinine was elevated at 1.6 mg/dL; LDH further elevated to 4,231 U/L; and albumin was 2.6 g/dL. Day 6 abdominal ultrasound showed renal echogenicity consistent with a medical insult, and a gall stone with surrounding fluid. Ceftriaxone was replaced on Day 6 with meropenem and vancomycin; metronidazole was added Day 7. On Day 7, the patient’s course steadily deteriorated with increasing abdominal distention and pain requiring narcotics, with new onset epistaxis. Abdominal X-rays showed paucity of gas without free air. Mental status was diminished, but the patient was arousable and verbal. A non-contrast head CT was normal. Elective intubation was performed to maintain respiratory status. CT scan of the chest/abdomen/pelvis showed marked hepatomegaly, moderate periportal edema, large bilateral pleural effusions and bibasilar atelectasis without pulmonary infiltrates. Bilateral chest tubes were required for the pleural effusions. Epinephrine and vasopressin (stopped after 5 days) were added to dopamine. Fever resolved on Day 7. On Day 8, the patient developed anasarca and had paracentesis Days 8 and 10 (culture negative) for ascites. Laboratory results showed progressive hepatic failure on Day 9 with secondary severe metabolic acidosis and coagulopathy: ammonia 72 μM/L, PT 24.7 sec, PTT 50 sec, fibrinogen 131, positive D-Dimers, AST 8,289 U/L, ALT 1,588 U/L, bilirubin 3.5 mg/dL, albumin 2.8 g/dL, and elevated lactate (101 mg/dL) with serum pH 7.17. Direct bilirubin was elevated when evaluated (Days 13, 17–20), with maximum of 9.3 mg/dL (indirect 12.6 mg/dL, total bilirubin 22.8 mg/dL) on Day 17. Skin biopsy (Day 14) of an erythematous confluent macular rash progressing to desquamation (onset Day 10) showed superficial perivascular and spongiotic dermatitis with eosinophils, consistent with either drug eruption or viral exanthem. By Day 18, there was worsening mucosal bleeding with frank blood from the paracentesis site and chest tubes. The patient was anuric by Day 20. Despite aggressive supportive care, the patient expired Day 20. Of note, this patient previously received ceftriaxone without hepatic toxicity or adverse reaction. Other concomitant medications included montelukast and ondansetron. Infectious evaluation included Influenza virus A&B Rapid DAA (Day 5); negative quantitative PCR for Epstein-Barr virus and cytomegalovirus (Day 6); Clostridia difficile toxin A&B DAA, Escheria coli 0157 screen, and stool adenovirus DAA (Day 8); negative respiratory culture (Days 8,10), non-reactive Hepatitis A IgM, Hepatitis B Core antibody, Hepatitis B surface antigen and reactive Hepatitis A IgG/IgM and Hepatitis B surface antibodies (Day 13, had history of Hepatitis A and B vaccines);and negative Acid Fast Bacilli, bacterial, viral, and fungal cultures of ascites, urine, trachea, liver and spleen (post mortem).

**Figure 1:**
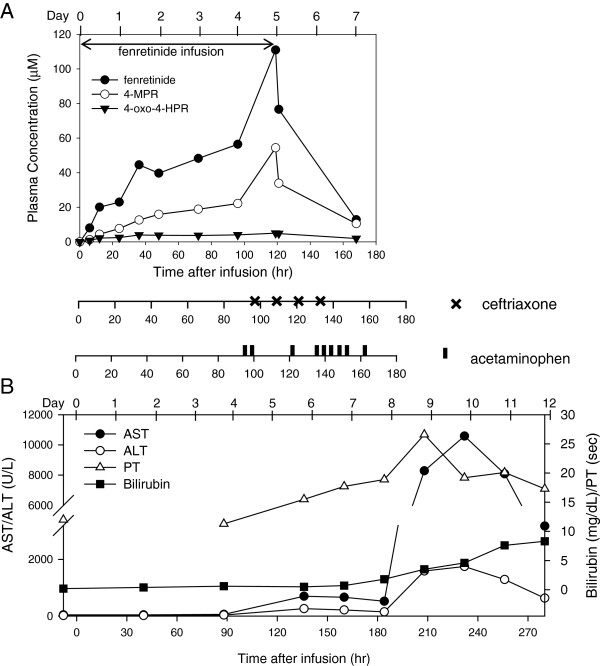
**Plasma concentrations of fenretinide and its metabolites, concomitant ceftriaxone and acetaminophen, and laboratory analysis in patient. A)** Plasma concentrations of fenretinide (closed circle) and the metabolites (4-MPR: open circle and 4-oxo-4-HPR: filled triangle) versus time after the initiation of infusion (hr) is plotted. Each dose of ceftriaxone (750 mg/dose), and acetaminophen (10 mg/kg for the initial dose and 15 mg/kg for subsequent doses) is indicated. **B)** Liver function analysis, AST/ALT, bilirubin (maximum for that date), and PT, are plotted on the same time scale. Due to the differences in values, the left Y-axis was used for plotting AST and ALT, and right Y-axis was used for bilirubin and PT. A PT value at 40 hours was not available.

Autopsy showed extensive hepatocellular damage and diffuse abdominal bleeding without evidence of tumor, infection, or allergic reaction. Liver findings included complete destruction of liver architecture with extensive hemorrhagic necrosis of liver parenchyma, marked bile duct proliferation, abundant hemosiderin consistent with cholestasis; no viral inclusions and negative immunohistochemistry for Herpes Simples virus 1 and 2, and cytomegalovirus. Post mortem skin biopsy showed subepidermal bullae and extensive upper dermal acute hemorrhage, likely a drug eruption since no evidence of an infection. Left ventricular papillary muscle necrosis was noted, likely contributing to circulatory failure and organ congestion. The autopsy was consistent with drug-related hepatotoxicity as the primary fatality-inducing event. Hepatic function, and fenretinide administration with plasma pharmacokinetics are summarized in Figure 1. The death was considered definitely related to fenretinide, but it remains unclear whether this was related to fenretinide-alone (e.g. plasma levels or total drug exposure) or to an interaction with other drugs that may have affected the elimination of fenretinide or acetaminophen (i.e., a hepato-biliary effect of ceftriaxone). The onset of fever with elevated LDH and ALT on Day 4, prior to starting ceftriaxone and acetaminophen, suggests that some hepatocellular injury may have occurred with fenretinide alone, but was likely exacerbated by these concomitant medications. The clinical generalized capillary leakage seen in this patient has been reported with infection, drug injury, multi-organ failure, and rarely with other retinoids [18].

While Grade 4 alkaline phosphatase elevations have occurred in children treated with fenretinide capsular and LXS oral power formulations, no alkaline phosphatase elevations occurred in the other patients on the NANT intravenous fenretinide study (Table 1). Grade 3 hypoalbuminemia in one patient receiving intravenous fenretinide and was reported in one patient receiving capsular fenretinide [13]. One fatal case of hepatic toxicity occurred in a seven year old female on the ANBL0321 capsular fenretinide phase II study at a dose of 2475 mg/m^2^/day for seven days, with onset 6 days after Course One, but extensive tumor infiltration in the liver on autopsy was considered to be the etiology. No other fatal hepatic toxicity has been reported on any other adult or pediatric fenretinide study using the capsule, oral LXS powder, or intravenous formulations [12,14,15].

In an adult intravenous fenretinide trial [15], several patients tolerated fenretinide plasma concentrations between 100–150 μM without hepatic toxicity. In this patient, fenretinide plasma concentrations gradually increased to ~60 μM at the end of Day 4. After acetaminophen and ceftriaxone were started on Day 5, fenretinide plasma concentrations increased steeply to ~110 μM (Figure 1A). Within two hours of stopping fenretinide, the fenretinide level dropped to ~80 μM and, after two days, was less than 20 μM (Figure 1A). Peak fenretinide concentrations in two other patients on this study were > 70 μM with minimal toxicity (Table 1). Considering the minimal hepatic toxicity in all fenretinide studies, and the tolerance of higher fenretinide plasma levels without hepatotoxicity, fenretinide alone may not be the cause of hepatic failure in the present case. Three other patients on the NANT 2004–03 study who received concomitant ceftriaxone all reported transient grade 1–2 hepatic toxicities during that course (Table 1). This may be due to inter-patient variation in biliary sludging of ceftriaxone. Three other N2004-03 patients received concomitant acetaminophen without hepatic toxicity; but no other patient received all three drugs concomitantly. Also, peak plasma levels were variable within a given dose, suggesting individual pharmacogenomics may play a role in the variation of plasma levels. . In the present case, the fenretinide plasma level increased sharply after ceftriaxone and acetaminophen were initiated. Retrospective attempts to measure acetaminophen levels in the fenretinide PK samples were not informative since the samples were beyond the stability duration of acetaminophen. Based on the abrupt decrease of fenretinide clearance, we hypothesize that acetaminophen clearance was also reduced due to the concurrent ceftriaxone. It is unclear whether acute liver failure would have been due to altered pharmacokinetics of acetaminophen or to hepatic glutathione depletion. Based on a previous report, intracellular glutathione levels are not related to fenretinide cellular cytotoxicity *in vitro*, suggesting that fenretinide may not affect intracellular glutathione [19]. In addition, N-(4-methoxyphenyl)retinamide (4-MPR) is the major metabolite of fenretinide and is a methylation (phase II metabolism) product eliminated without further modification. Therefore, it is less likely that acetaminophen and fenretinide competed for glutathione - competition for UGT-mediated glucuronidation is more likely. This case suggests that the biliary sludging effect of ceftriaxone may have interfered with the elimination of fenretinide and acetaminophen, resulting in an unexpected adverse and severe acetaminophen toxicity. However, ibuprofen-related hepatic failure cannot be ruled out as it is reported that therapeutic doses of ibuprofen as a single agent can result in acute liver failure [20].

After this event, NANT 2004–03, and related adult trials, PhI-42 (NCT00104923) and PhI-54 (NCT00387504), were amended to prohibit ceftriaxone or acetaminophen for 24 hours prior to the fenretinide infusion and for 24 hours (ceftriaxone) or 48 hours (acetaminophen) after fenretinide infusion completion, with ibuprofen recommended (if needed) for fever during the fenretinide infusion. After enrolling two patients following this amendment, the NANT 2004–03 trial closed secondary to insufficient drug supply prior to determining an MTD.

## Conclusion

This clinical case reports lethal hepatotoxicity in a patient concurrently receiving intravenous fenretinide, ceftriaxone, and acetaminophen. The event is suspected to result from an unexpected drug interaction between these three agents resulting in hepatic acetaminophen toxicity. Although the prevalence of cholestasis with ceftriaxone usage is relatively high, the potential drug interaction with these concomitant medications has not been previously reported. Concomitant use of fenretinide, ceftriaxone, and acetaminophen should be avoided.

## Consent

Written informed consent was obtained from the patient’s guardian for publication of this Case Report and any accompanying images. A copy of the written consent is available for review by the Editor-in-Chief of this journal.

## Abbreviations

NANT: New Approaches to Neuroblastoma Therapy; LDH: Lactate dehydrogenase; AST: Aspartate aminotransferase; ALT: Alanine aminotransferase; PT: Prothrombin time.

## Competing interests

The Children’s Hospital Los Angeles (CHLA) holds patents and/or patent applications on intravenous fenretinide (the study drug). CHLA and co-inventors of the study drug, Drs. BJM and CPR, Texas Tech University Health Sciences Center, Lubbock, TX, may potentially benefit financially from the development and future use of the study drug. JGV and AM are on the medical staff at CHLA, and SC is a CHLA employee, and may indirectly benefit from the development of the study drug.

## Authors’ contributions

MHK analyzed pharmacokinetic samples of the patient and wrote the manuscript; JGV was the primary oncologist of the patient; KKM and JGB were co-chairs of NANT 2004–03; AM was on the study committee of NANT 2004–03; JGV, AM, KKM, SG, RS, MMA, CPR, and BJM provided substantial intellectual contributions to the study as clinical trialists, biostatisticians, or a pharmacologist. JGV, AM, KKM, SG, and SC were part of the NANT study monitoring committee for N2004-03; SC was the research nurse for N2004-03. JGV, BJM, and CPR participated in writing the manuscript. All authors read and approved the final manuscript.

## Authors’ information

MHK, pharmacologist: Associate Professor and Director of Clinical Pharmacology Laboratory, Cancer Center, TTUHSC, JGV, pediatric oncologist: primary care physician of the patient presented in this manuscript. JGB, pediatric oncologist: participating physician of the phase I study, Study chair for other clinical trials for COG and NANT. KKM, pediatric oncologist: Senior Editor of Clinical Cancer Research, SG, Biostatistician: COG, NANT, and California Cancer Consortium biostatistician. RS, biostatistician: COG and NANT biostatistician, SC, RN and CRA: for NANT operation center. MMA, PhD in pharmacologist: Director of Clinical Pharmacology Laboratory, Mayo Clinic, CPR, MD/PhD: Director of Cancer Center, TTUHSC, BJM, pediatric oncologist (MD/PhD): associate professor, TTUHSC.
